# Manic temporality

**DOI:** 10.1080/09515089.2018.1502873

**Published:** 2018-08-08

**Authors:** Wayne Martin, Tania Gergel, Gareth S. Owen

**Affiliations:** a School of Philosophy and Art History, University of Essex, Essex, UK; b Institute of Psychiatry, Psychology, and Neuroscience, King’s College London, London, UK

**Keywords:** Bipolar, Edmund Husserl, Eugène Minkowski, Ludwig Binswanger, phenomenology, protention, time-consciousness

## Abstract

Time-consciousness has long been a focus of research in phenomenology and phenomenological psychology. We advance and extend this tradition of research by focusing on the character of temporal experience under conditions of mania. Symptom scales and diagnostic criteria for mania are peppered with temporally inflected language: increased rate of speech, racing thoughts, flight-of-ideas, hyperactivity. But what is the underlying structure of temporal experience in manic episodes? We tackle this question using a strategically hybrid approach. We recover and reconstruct three hypotheses regarding manic temporality that were advanced and modeled by two pioneers of clinical phenomenology: Eugène Minkowski (1885–1972) and Ludwig Binswanger (1881–1966). We then test, critique, and refine these hypotheses using heterophenomenological methods in an interview-based study of persons with a history of bipolar and a current diagnosis of acute mania. Our conclusions support a central hypothesis due to Minkowski and Binswanger, namely, that disturbance in the formal structure of temporal experience is a core feature of mania. We argue that a suitably refined variant of Binswanger’s model of disturbance in manic protention helps to explain a striking pattern of impaired insight and impaired reasoning in manic episodes.

## Introduction

Symptom scales and diagnostic criteria for mania are peppered with temporally inflected language: increased rate of speech, racing thoughts, flight-of-ideas, hyperactivity. Moreover, a growing body of research lends support to the hypothesis that mania involves some kind of disturbance in the character of temporal experience. One study found that, in comparison to healthy controls, persons in acute manic episodes reported an accelerated “flow of time” and overestimated duration in a variety of duration-judgement tasks (Bschor et al., 2004). A second study found that persons with mania tend to remember time intervals as having been shorter than they actually were (Mahlberg, Kienast, Bschor, & Adli, 2008). These recent findings support earlier controlled studies which reported on accelerated subjective time experience in mania (Mezey & Knight, 1965; Tysk, 1984). Drawing on a review of existing literature, Bschor and colleagues sought to clarify the extant experimental vocabulary in this area of research, defining “time experience” as “the subjective experience [of] how fast or slow time is *passing*” and defining “time sense” to include “all aspect of experiencing time *flow* and judging time *spans*” (Bschor et al., 2004, pp. 222–223, emphasis added).

In what follows, we seek to build upon these recent research findings by exhibiting a distinctive feature of temporal experience in mania. In order to do so, we turn to the tradition of *clinical phenomenology*. Drawing on insights from that tradition, we bring into focus an aspect of temporal experience in mania that has not been thematized in recent empirical research and which is distinct from the experience of passing time, time flow, or judgement of duration.

Temporal experience has been one of the enduring concerns of the phenomenological movement since its inception, and some of its most important research results have emerged in this area. But while the celebrated investigations of Bergson, Husserl, and Heidegger regarding temporal experience have been extensively discussed and debated (see, e.g. Blattner, 1999; Guerlac, 2006; Kortooms, 2002; Miller, 1984), what has attracted less attention are the findings of a group of pioneering clinicians who sought to use the phenomenological investigation of time-consciousness to develop a better understanding of the structure of temporal experience in their patients. In what follows our aim is to recover and extend this tradition of clinical phenomenological investigation of temporal experience, with particular attention to the experience of acute mania. In doing so, we adopt a strategically hybrid approach, first reconstructing the accounts of manic temporality proposed by leading twentieth century clinical phenomenologists, then testing their theories against empirical evidence drawn from a clinical study.

We proceed as follows. We begin by identifying three key hypotheses regarding manic temporality that were first formulated by Minkowski (Section 1). We then reconstruct and assess two phenomenological models for those hypotheses developed by Binswanger (Sections 2–4). In the final sections of the article (Sections 5 and 6), we test and refine Minkowski’s hypotheses and Binswanger’s models using heterophenomenological data from an interview-based study of temporal experience in the acutely manic phase of what is clinically described as “bipolar disorder” or “bipolar I.” Our conclusions (Section 7) support a central hypothesis due to Minkowski and Binswanger, namely, that disturbance in the formal structure of temporal experience is a core feature of mania. We argue that a suitably refined variant of Binswanger’s model of disturbance in manic protention helps to explain a striking pattern of impaired insight and impaired reasoning in manic episodes. Our results bring into focus a structure of Husserlian protention that has not been generally recognized, which is displayed most vividly under conditions of pathology, and which suggests a deep connection among temporality, inductive reasoning, and self-knowledge.

## 1. Minkowski’s three hypotheses

As an initial benchmark in surveying the tradition of clinical phenomenology, we turn to a seminal case study reported by Minkowski in 1923. The case does not itself involve mania, but it matters for our purposes nonetheless because it records rather vividly the initial framing of a cluster of insights about temporality and mood disorder whose validity we propose to assess. Minkowski recounts an extraordinary episode during which he served as the personal physician to a 66-year-old man suffering from a severe form of depression. The man was besieged by feelings of guilt, and lived in continual anxiety both about his own imminent torture and execution and the execution of members of his family. His persistent and distressing delusions included the conviction that all the waste and refuse of the world was to be pumped into his stomach.

Minkowski’s relationship to this patient was unusual. His duties as personal physician were so arranged that he lived with the man continuously, day and night, for a period of 2 months, apparently in quite intimate quarters.^1^ It was during this extended close interaction and observation that Minkowski hit upon what would become his principal hypothesis. His training had taught him to probe the man’s condition by first examining the specific content of the delusional experiences, and then endeavoring to trace them back to specific disorders of perception or judgement.^2^ But over the course of their shared residence, Minkowski came to a different conclusion. “Where,” he asked, “is the discordance between [the patient’s] psyche and [my] own?” (Minkowski, 1923, p. 131). His answer: the patient’s most fundamental disorder was neither perceptual nor judgmental but *temporal*. And the most important difference between doctor and patient pertained not to the *content* of the patient’s delusions but to the *form* of his temporal experience.^3^

Intriguingly, one clue that led Minkowski to this hypothesis concerned a distinctive impairment of his patient’s reasoning abilities: each day, in a state of considerable anxiety and fear, the man would insist that his execution would take place that night. Minkowski reports on his own reaction:

[At first] I consoled myself with the thought that, come the morning, he would see that all his fears had been in vain. However, the same scene was repeated the next day and the next, until after three or four days I had given up hope, whereas his attitude had not budged one iota. What had happened?…. I, as a normal human being, had rapidly drawn from the observed facts my conclusions about the future. He, on the other hand, had let the same facts go by him, totally unable to draw any profit from them for relating himself to the same future. I now knew that he would continue to go on, day after day, swearing that he was to be tortured to death that night, and so he did, giving no thought to the present or the past. (Minkowski, 1923, p. 132)

Already with this one case report we encounter a number of the themes that will concern us in what follows. Minkowski’s patient suffers from a condition in which his thought processes and affective life fail to track the realities of his situation. That condition itself involves the collapse of a fundamental component of ordinary human reasoning – what Minkowski calls “the tendency to generalize,” or what we might call *inductive reasoning*. And all this, Minkowski boldly proposes, is rooted in “a profound disorder in his general attitude toward the future.” Minkowski hazards several descriptions of the underlying disturbance in the patient’s lived experience of time: “That time which we normally integrate into a progressive whole was here split into isolated fragments”; “[the] carry-over from past and present into the future was completely lacking in him”; “[he] was completely lacking … [a] propulsion toward the future”; “the future was blocked” or “shut off” (Minkowski, 1923, pp. 132–138).

The mature statement of Minkowski’s view is found in his major study of 1933: *Lived Time*. In a brief but important passage, Minkowski applies his hypothesis to mania.

In my estimation, the structural analysis of the person in a state of manic excitement will have to be undertaken in this direction, and this all the more so because it allows us to view manic excitement from the same point of view as melancholia, namely, *as a disorder relevant to unfolding in time, or if you wish, as a manifestation of a mental subduction in time*. (Minkowski, 1933, p. 296, emphasis added)

Minkowski’s hypothesis is that mania has its roots in the temporal structures of human experience, and specifically in an impairment in the way that time “unfolds” – that is, the way in which temporal experience is articulated into a past, present, and future. Borrowing a term from Mignard (1924), he describes the underlying disorder as a “subduction” in time – that is, as a “modification to an inferior level.”^4^ As to the specific form that subduction takes in mania, Minkowski proposes the following description:

A person in a state of manic excitement *lives only in the now*, and his contact with the environment is restricted to the now; *he has no present any more*, since in general he no longer experiences “unfolding in time.” (Minkowski, 1933, p. 294, emphasis added, translation altered)

Following Husserl, Minkowski distinguishes between a temporally and hermeneutically thin *now* and a thick, expansive, and hermeneutically rich *present*. He argues that when a person’s lived future is lost or distorted under conditions of mania, the result is a “subduction” in temporal experience. In Minkowski’s Bergsonian terms: “There is no longer any lived duration [*durée*]”; the manic individual becomes “the plaything of the now, always variable, changing from one instant to the next”; he lives “in the grip of the now, in which he exists and out of which he is incapable of creating a present” (Minkowski, 1933, pp. 294–296).

We need finally to take note of one further claim advanced in Minkowski’s brief remarks about mania. Notice that in the passages just cited, Minkowski claims not only that mania involves living “only in the now,” but also a resulting restriction in “his contact with the environment [*son contact avec l’ambiance*].” In elaborating this idea, Minkowski writes of “the particular shrinking [*rétrécissement*] which the manic person’s vital contact with reality undergoes, which, without completely annihilating it, renders this contact particularly superficial” (p. 295).

Minkowski himself never provided a detailed development of these proposals, and many of his formulations are suggestive without being precise. But he nonetheless framed a trio of hypotheses of considerable importance for subsequent research. To summarize: (1) Disturbance in the formal structure of temporal experience is a core feature of mania. (2) Persons in states of mania “live only in the now,” no longer experiencing the usual unfolding of time into a complex present, past, and future. (3) Disturbance in the temporal structure of experience “limits contact with reality” for the person undergoing a manic episode.

## 2. Binswanger’s first model

Minkowski described his three hypotheses about mania as mere “suggestions” (Minkowski, 1933, p. 294). But his core hypotheses were later developed by his Swiss contemporary and friend, Ludwig Binswanger, who credited Minkowski as being “the first to introduce phenomenology into psychiatry for practical purposes” (Binswanger, 1946, p. 231). Unlike Minkowski, who wrote about manic experience only in passing, Binswanger devoted two books to the topic; they were separated from each other by nearly 30 years. He also approached the topic with something that Minkowski seems to have lacked: detailed knowledge of the phenomenological research on temporal experience undertaken by Husserl and Heidegger.^5^ Indeed, we can usefully treat Binswanger’s work in this area as an attempt to provide a model for Minkowski’s hypotheses, using resources drawn directly from Husserl and Heidegger.^6^ In the end, Binswanger provided not one but two such models – one drawing primarily on Heidegger and one drawing mainly on Husserl. In this section, we examine the first model, returning to the second model in Section 4.

Binswanger’s *Über Ideenflucht* (1933)^7^ was published as a book in the same year as Minkowski’s *Lived Time*. Its focus was the so-called “flight-of-ideas” that has long been one of the presentations most closely associated with mania. Theoretically, it was profoundly shaped by Heidegger’s *Being and Time*, which had been published in 1927 (and which received only a footnote mention in Minkowski’s study). The broad strategy of Binswanger’s book was to use certain core concepts and strategies from *Being and Time* in order to describe and articulate manic experience. For our purposes, three ideas are central: the claim that there is a distinctive, world-disclosive mood [*Stimmung*] associated with manic episodes, the claim that this mood has its own distinctive *temporal structure*, and the thesis that the temporal disturbance at work in mania can be understood as a narrowing or “shriveling” in the way in which “temporality temporalizes itself.” We consider each of these points in turn.

Binswanger’s point of entry for this early investigation was the phenomenon of *manic optimism*. He distinguishes two broad forms of optimism, which he refers to as “primary” and “secondary” optimism. The defining feature of secondary optimism is its dependence on some belief or “positing” of one kind of another. One of Binswanger’s examples is Odysseus, whose optimism about getting home to Ithaca derives from his explicit belief that Athena will protect him in fulfillment of her promises. Panglossian optimism rests on positing that this is the best of all possible worlds. From these various forms of secondary optimism, Binswanger distinguishes primary optimism as a “*Stimmung*” – as a mood or attunement. Unlike that of Odysseus or Pangloss, it does not rest on any conviction, posit or belief. He coins the term “*Stimmungsoptimismus*” (optimism-as-mood) to distinguish it (p. 57).

Applying Heideggerian principles, Binswanger holds that the manic mood of primary optimism is both “world-disclosive” and temporally structured. According to Heidegger, to be in a particular mood is to find oneself in a world with a distinctive texture.^8^ Accordingly, Binswanger’s analysis of manic experience begins by trying to articulate the texture of the world of manic optimism. The terms in which he does so are often metaphorical. The manic world is “all-rosy,” cloudless, bright, and “wide-open” (p. 58). Even at this first stage of the phenomenological articulation, temporal features enter in. This is hardly surprising, insofar as optimism would seem to be an essentially temporal phenomenon, implicating a sense for a future with a distinctive valence. The manic individual, Binswanger observes, always “plans, hopes or expects something pleasing,” and has the sense of an unlimited horizon of time. Even death, if it is considered at all, is encountered as a gateway to a further existence (p. 58). So the manic world is temporally infinite.

The next stage of Binswanger’s investigation again follows a Heideggerian path. Having described these features of the manic mood and its world, he sets out to identify the underlying structures of temporal experience associated with it (pp. 153–162). The description he offers is closely modeled on a largely neglected section of *Being and Time*, in which Heidegger describes the distinctive temporal rhythm of what he calls “curiosity.”^9^ The curious individual whom Heidegger describes is characterized by a superficial, highly distractible, enthusiastic attraction to whatever proximately presents itself. Adapting a comment from Rilke, Binswanger describes the “tempo” of manic curiosity as involving a form of leaping [*springen*], tumbling [*taumeln*], and sliding [*gleiten*] from one thing to another. Following Heidegger, he emphasizes the crucial consequence. Mania brings with it what Heidegger calls *Aufenthaltlosigkeit*: the manic individual never lingers or “tarries” in a situation but is ever and always leaping off to something new.^10^ Binswanger is particularly struck by Heidegger’s description of a condition of the curious individual as being at once “*überall und nirgends*” – everywhere and nowhere. So for Binswanger, mania is effectively assimilated to an extreme case of what Heidegger calls inauthenticity.^11^

The final stage in Binswanger’s early analysis is also an attempt to follow Heideggerian principles. For Heidegger, the fundamental structures of our existence have their deepest source in the distinctive ways in which, in his notorious jargon, “temporality temporalizes itself.” The primitive or “original” structure of temporal experience becomes articulated or “unfolded” into the familiar, orderly, sequential structures of past, present, and future. Binswanger’s thesis is that the core phenomenon at work in mania is a disturbance in this temporal articulation. In mania, Binswanger claims, “the structure of temporalizing is shrivelled [*geschrumpft*] and narrowed to mere enpresenting [*Gegenwärtigen*]” (p. 162).

Even with this much of Binswanger’s story in view, it should already be clear that and how Binswanger’s position in 1933 provides a Heideggerian model for Minkowski’s hypotheses.^12^ Minkowski’s first hypothesis was that disturbance in the formal structure of experience is a core feature of mania; Binswanger tries to specify just what that disturbance is. Minkowski claimed that the manic individual lives “in the grip of the now”; Binswanger traces this back to a fundamental narrowing or “shriveling” (p. 162) in the unfolding of temporal experience. Minkowski claims that one result of these temporal disturbances is a loss of vital contact with reality; Binswanger describes manic *Aufenthaltlosigkeit* [restlessness], which he identifies as “the most extreme counter-phenomenon” [*äußerste Gegenphänomenen*] to what Heidegger calls the *Augenblick* – the experience in which we are able to grasp a situation for what it is and respond to it resolutely (p. 159).^13^

Binswanger’s early model of manic temporality is fascinating, not least as a vivid trace of the impact of *Being and Time* upon an “early adopter” from the world of clinical psychiatry. But it is not without its difficulties. Two in particular merit comment at this stage. First, although Binswanger relies heavily on Heidegger’s theory (and jargon!) in elaborating his model, it is not clear that the resulting position makes sense in Heideggerian terms. Specifically, it is not clear to what extent the “shriveling” or “narrowing” of manic temporality is consistent with Heidegger’s theory of temporal experience. Here, alas, Binswanger does not say as much as one might hope to specify exactly what the “shriveling” and “narrowing” consists in. But if it is understood to mean that there could be a form of meaningful presence *without an experience of past or future*, then this would seem to be at odds with some of Heidegger’s most fundamental doctrines: his thesis about the existential priority of the future, and most importantly, his thesis about the original unity of the three temporal ecstases. For Heidegger, in short, the experience of a meaningful present is fundamentally dependent on an experience of a richer temporal structure that includes futurity.^14^

A second difficulty is more internal to Binswanger’s own commitments – a potential tension between his starting point and his final position. Here we have to recall that Binswanger’s point of entry for the phenomenology of manic experience was manic optimism. Optimism itself would seem to be an intrinsically temporal mood; it is constituted in part by an orientation toward good things to come. Moreover, as we noted, Binswanger himself provides an articulation of some of the distinctive temporal structure of the world disclosed by *Stimmungsoptimismus* [optimism-as-mood] – including the sense of temporal openness toward an unbounded future. So there is at least a prima facie problem in accommodating all this with the ultimate conclusion about a form of temporality that is “shriveled” or narrowed to mere presence.

Neither of these problems are necessarily insuperable. But in order to resolve them we would need something that Binswanger’s 1933 book does not provide: a more detailed analysis of the specific structure of the experienced present that predominates in mania. Thirty years later, Binswanger attempted to provide exactly that. But this time he saw the need to supplement his Heideggerian strategies with resources drawn from Husserl’s theory of time-consciousness.

## 3. A very brief comment on protention and retention

Before considering Binswanger’s second model of manic temporality, we need to revisit Husserl’s core concepts of retention and protention. Husserl’s landmark 1905 lectures on what he called “internal time-consciousness” articulated a distinction between an instantaneous “now” and what, following William Stern, he refers to as *Präsenzzeit* – the “lived present” or “presence time.”^15^ In unpacking the phenomenological structure of the experienced present, Husserl identified the twin phenomena of “retention” and “protention.” Retention and protention are temporally inflected forms of intentional experience. But Husserl insists that their intentional structure differs from that of the more familiar phenomena of remembering and expecting.

In the standard case of memory, as Husserl understands it, we “recollect” or “recapitulate” a past event or episode. In doing so the event or episode “comes to mind.” That is, it *becomes present once again* – whether because we intentionally *bring* it to mind, or because it happens to “spring to mind.” Husserl introduces a key piece of technical terminology to refer to this phenomenon of “becoming present” – *Vergegenwärtigung*. The term defies any easy translation; we resort to James Churchill’s neologism “presentification” (Husserl, 1964, p. 36n). The key point, as applied to the past, is that what Husserl calls secondary memory (recollection) involves presentification of some sort: what is past is “made present” for conscious attention. Retention is different. In retention, we neither recollect nor recapitulate the past event, and we do *not* experience the past content as present once again. In Husserl’s provocative shorthand: *I directly perceive the past* (Husserl, 1950, vol. X, pp. 39, 79).

Husserl exhibits the phenomena he has in mind with reference to his example of the melody. If someone plays a simple tune with one hand on a piano, but then abruptly stops midway through, we experience a *retention* of the note that has just played, experiencing it not as present but as past. At the same time we have a protentive experience, in a characteristic modality of partly determinate indeterminacy, of the as-yet-still-future note to follow. Crucially, we do not hear all of this in the same mode; if we did then we would hear either a chord or a cacophony. Husserl argues that these retentive and protentive experiences are the primal manifestation of time in our experience, that they are essentially “non-presentifying,” and that they are both distinct from and conditions upon the more familiar temporal experiences of reproductive memory and expectation.

The foregoing summary is a fairly standard synopsis of Husserl’s position, even if commentators differ on particular details or points of emphasis. But at this point, we reach an important fork in the road. Husserl’s reliance on the musical example has led many readers and commentators to assume that retention and protention pertain narrowly and specifically to the experience of the only-just-past and the about-to-occur. Zahavi, for example, characterizes a protention as “a more or less indefinite intention *of the phase of the object about to occur*” (Zahavi, 2003, p. 83, emphasis added). The assumption that retention and protention extend *only* to the temporally proximate is both reflected in and reinforced by the history of (mis)translating Husserl’s “*Präsenzzeit*” [presence-time, lived presence] as “the specious present” – a term which has been used in psychology specifically in connection with the experience of concurrence.^16^ It is not entirely clear whether Husserl himself intended to restrict the use of “retention” and “protention” to the temporally proximate. But whatever Husserl’s own position on the matter of definition, it will be crucial for our purposes to distinguish two discrete commitments of his substantive theory. The first is the identification of retention and protention as *sui generis* “non-presentifying” intentional acts directed at past or future stimuli, events, or phases of temporally extended objects. The second is the particular *application* of these concepts in the interpretation of the experience of the “just-now-passed” and the “just-about-to-occur.” In seeking to understand and develop Binswanger’s later theory of manic temporality, we will need to disentangle the two commitments.^17^

## 4. Binswanger’s second model

With this distinction in hand, we can now turn to Binswanger’s second model, which he develops in his 1960 book *Melancholia and Mania*.^18^ The book takes the form of a series of studies intended to provide a unified theory of the two phases of what we would now call *bipolar I*, and which Binswanger describes as “the manic-depressive antinomic” (p. 113). The two central concepts for our purposes are, first, the idea of the “construction of the present” [*Aufbau der Gegenwart*], and, second, the idea of Husserlian protention and retention as “*maßgebenden intentionalen Fäden”* – roughly: *normative intentional threads*. We address these two concepts in turn. The first concept, the *“Aufbau* of the present,” is a natural development of insights shared by Husserl, Heidegger, and Minkowski. If the now is temporally simple while the lived present is temporally complex, then we need an account of the temporal constitution of the present – the way that it is “*built up*” or “constructed.”^19^ In providing an account of that “*Aufbau*” [construction], Binswanger describes protention and retention as the “intentional threads” out of which the temporal “fabric” is woven (p. 96).

It should be clear that we are once again here in the domain of metaphor. Binswanger thinks of lived time as a kind of temporal “fabric” or “weave” [*Gewebes*] which makes it possible for temporal objects to appear and indeed mediates our whole relationship to our environment. Protention and retention are then understood to be the major threads (the warp and the weft, so to speak) out of which that fabric is woven. Crucially, Binswanger describes these threads as *maß-gebenden* – that is, as “establishing the measure” or as “norm-giving.”^20^ The substantive thesis is then that temporal experience is “built-up” or constructed in part from a set of protentions and retentions against which experience is measured or assessed. In affective disorders, Binswanger claims, this “weaving up” of the present becomes distorted.

This is a tantalizing proposal, and one that points toward the possibility of filling the lacuna we found in his earlier model. If we could provide a perspicuous account of this temporal *Aufbau* [construction, structure] and its disturbances, we would be on our way to an articulation of the phenomenological substructure of temporal experience in mania. That is the good news. The bad news is that the promise is never properly fulfilled. One persistent problem is that Binswanger’s writings on this topic never break free from these first metaphorical formulations. Binswanger offers a number of suggestive elaborations of his governing metaphor of the “fabric,” “weave,” and “threads,” but the metaphor itself is never fully cashed.

The more serious problem for present purposes is that Binswanger operates with a number of competing deployments of the metaphor; these different versions themselves pull in rather different directions and are not obviously consistent. His most frequent elaboration (and the “official story”) is that in conditions of manic excitation the weave of the temporal fabric undergoes a kind of “loosening” [*Auflockerung*] (p. 96). Note that if we take this metaphor seriously, we would have to say that the normative intentional threads of protention and retention remain; they are just not woven together as tightly as in other configurations of experience. One result might be that certain kinds of extended temporal objects simply cannot be experienced; they “slip through the gaps” in the weave of our temporal fabric, which lacks the degree of continuity needed to “capture” them. But in other passages, Binswanger claims that in mania the temporal threads are “torn up [*zerreißen*] into fragments,” (p. 95) “retreat [*Zurücktreten*] or indeed disappear [altogether” (p. 115). On this application of the metaphor, the collapse of temporal experience would be more thoroughgoing. If the threads disappear then surely the fabric goes with it.

But alongside these first two deployments of his metaphor, Binswanger on one occasion makes use of a third. Interestingly, the third deployment is the one that is most tightly connected to one of Binswanger’s case studies, *The Case of Olga Blum*. As is often the case with Binswanger’s case studies, he sketches the basic facts of the case and then intensely focuses on an illuminating moment from one particular episode. With Olga Blum, the episode involves reading Goethe. At one point during her stay at his sanatorium, Binswanger gives Olga a copy of *Faust* and invites her to read it. When later asked for her reaction, her response is: *I am lucky that Goethe lived before me; otherwise I would have had to write it!* (p. 99).

In reflecting upon this response, the feature that Binswanger finds most significant is not so much Olga Blum’s relief at not having to write *Faust* herself, but rather the characteristic form of manic optimism reflected in her confidence that *she could indeed write it herself if she had needed to*. Binswanger elaborates on this optimism in terms of its protentive structure:

The protentive moment emerges from the whole sentence in which Olga Blum (both) expressed her relief that the prior existence of Goethe relieved her of the effort of having to write a *Faust* like his, while at the same time harboring no doubt that she would succeed at such a task. This is *the protention that hangs in the air*, since all retentive moments are missing, on which she would be able to build (p. 101, emphasis added).

Notice here a third deployment of Binswanger’s governing metaphor. What he ascribes to Olga Blum in this passage is not a loosely woven temporal fabric, or a form of experience in which the protentional and retentional threads are *zerreißen*. For Olga Blum a distinctive form of manic protention is indeed at work, but it “hangs in the air,” without being interwoven with the correlate retentions.

## 5. Six patterns

We turn now to the second strand of our strategically hybrid approach. While the proposals developed by Minkowski and Binswanger are rich and suggestive, and while they were grounded in extensive clinical experience, they also suffer from intrinsic limitations. As we have seen, their positions are often articulated in metaphors that are difficult to cash, and they suffer from ambiguities and prima facie inconsistencies that are difficult to resolve. Binswanger’s second model is in many ways the most developed theory from this tradition, and was the fruit of many decades of clinical experience and phenomenological research. But even here, we encounter multiple deployments of key metaphors without the evidence that would allow us to choose among them. In order to overcome these limitations, and in order to assess the models we have documented, we introduce empirical evidence from our interview-based study of manic temporality. Our presentation of findings here is of necessity selective; for present purposes we focus on those that have direct bearing on Minkowski’s hypotheses and Binswanger’s models.

We begin with a few words about the design of the study. The participants were 12 individuals selected for histories of bipolar disorder, being acutely manic at the point of the interviews, and being effective communicators of their manic experience. The study received research ethics approval and the rules of the *Mental Capacity Act* (2005) were followed.

We employed the technique of “second-person phenomenology” on which we have reported in earlier work (Owen, Freyenhagen, Hotopf, & Martin, 2015; Owen, Freyenhagen, & Martin, 2018; Owen, Freyenhagen, Martin, & David, 2017). We approached the research participants as collaborators, and as informants as to the character of their own manic experience. In a series of approximately 1-hour, semi-structured interviews, participants were invited both to report on their ongoing experiences at the time of the interview and to reflect on earlier experiences of manic episodes, as well as the possibility of future ones. The interviews were conducted by an experienced clinical psychiatrist (GO) and were organized around a series of open questions, with opportunities for follow-up and discussion between interviewer and participant.^21^

In keeping with Dennett’s proposals for “heterophenomenology” (Dennett, 1992), we treated participant reports as primary data to be interpreted; we did not assume that they were always *reliable* informants. The interviews were recorded, transcribed, and subsequently “coded” using iterative tagging (Charmaz, 2006), undertaken collaboratively by the three co-authors. The recordings, coded transcripts, and contextual information were then submitted to close interpretative scrutiny. We used the data from the interviews as evidence from which hypotheses regarding participant experience could be generated. We then probed these hypotheses both in subsequent interviews and in further analysis of recordings and transcripts. We used techniques of *grounded theory* (Charmaz, 2006; Glaser & Strauss, 2017) to assist in the construction of higher-order codes and in the thematization of our data. Excerpts from the clinical interviews cited in this article are drawn from a large corpus of interview data.

So what did we find? We identified six patterns in the interview data that related either directly or indirectly to our core question about temporal experience in mania. Before turning to the patterns, however, it may be helpful to review a few responses to one of our ice-breaking questions. We asked participants how they would explain their experience of mania to someone who had no experience of the condition. Here are three indicative responses, at least two of which, as it happens, appeal to scenes from well-known films.

1. BP7: It’s bright, it’s very bright. It’s as bright as the sun is…. The light is part of me…. I’m part of the circle of life; I’m part of the energy of life.
2. BP6: It sounds very weird and stupid but – *Charlie and the Chocolate Factory*. You know when the elevator smashed through the roof?…. Reach the peak and it’s … through the roof, through the ceiling…. Not nasty. It’s like it’s being free…. He looked down and he could see everything, and everything was his.
3. BP10: I just love every moment, like you know, the blue of the sky, the white of the crowds, the green of the trees, you know…. It’s like Julie Andrews in the opening of *The Sound of Music* where she’s spinning her arms around and just singing: “The hills are alive…” [*Laughs*].

With this by way of background, we turn now to review the six relevant patterns. First, all participants were able to report on past manic episodes, and many were able to do so in considerable detail.

(4) BP6: Er…. I was with a girl, we was in love and she was great. We smoked a lot of weed, we drunk a lot of wine, we worked hard. Er… so much so that we rarely cooked, we was out nearly every night having a meal. A very nice life. But it only lasted like a year, and then maybe we had one spliff too many, and then…. We had our first sleepless night and I woke up and I felt sort of weird. I was like sort of up there in the corner, I looked down and I could see myself sleeping on the bed next to her. I don’t know what happened, and that was it. And then the next thing I knew, I was naked, walking up the shops for a pint of milk and a packet of fags…. I thought it was alright. I thought “Oh I can do what I like, and why not?” … and the next thing you know I got arrested for indecent exposure, and the police arrived, and the landlord arrived, and then I was in hospital.

Second, participants were able to make reference to concrete future plans.

(5) BP9: I’ve just got to make sure I pay my TV license this week.

Third, many participants were able to report in some detail on the warning signs of an imminent manic episode.

(6) BP2: I always know when I’m going to be manic.
Interviewer: You always know?
BP2: Yeah. I always know because I become happier, I become more inclined to listen to loud music, I tend to buy things, I tend to be jolly on the whole except when somebody gives me a problem, then I become very irritable.
Interviewer: So you know? You know what the signs are?
BP2: I’ve a got a big insight, and it’s normal. I’m not at the stage where it’s the first time in my life that I had a manic episode. I’ve had manic episodes for 35 years.

Particularly in light of these first three patterns, a fourth pattern was particularly striking: all of the participants denied that they were currently in a manic state, despite being hospitalized with a diagnosis of mania.

(7) BP4: When it comes to bipolar, I’m the king of bipolars.
Interviewer: Would you say you were in a manic phase now?
BP4: No.
Interviewer: What phase are you in now, would you say?
BP4: Just in a level phase.

A fifth pattern concerned the possibility of *future* episodes, which the participants almost all denied.

(8) BP9: I can’t even imagine one [future manic episode] … I’m through it. I’ve not peaked and gone down. I’m in a different arena. This is a new game now.
(9) Interviewer: So what you’re saying is that all the issues about mania, and managing mania, in the past are…
BP4: Fizzling out.

The sixth pattern emerged in response to more open-ended questions about the future.

(10) Interviewer: When you look to the future, do you see pain and suffering, or do you see possibilities?
BP4: No. I see freedom and liberation…
(11) Interviewer: So when you think about the future now, how does the future seem to you?
BP9: It’s great, it’s golden, it’s wonderful. It’s going to be full of all the things that I could potentially have, create for myself…. I know that my life will be full of abundance [clicks fingers] if I keep this synchronistic approach.
(12) Interviewer: Do you have worries about the future, or do you feel that the future’s …?
BP5: No. I don’t fear about the future, the future will take care of itself.
(13) Interviewer: What can you see if you look into the future?
BP6: Palm trees, whitewashed buildings, hot sun. Anywhere, like that. But not just that, you know. Places where I haven’t been.

This distinctive attitude toward the future, both highly positive and yet also largely unspecific, was the single most prevalent temporal theme in the interviews.

What light do these patterns shed on Minkowski’s hypotheses? We suggest that our data effectively rebut Minkowski’s second hypothesis, if that is understood to mean that persons in conditions of mania live without awareness of past or future. The participants in our study were severely manic at the time of these interviews, but they nonetheless reported and reflected in rich detail on their own past, and in both mundane and extraordinary ways on their future. They did not “live only in the now.”^22^ This same evidence casts doubt on Binswanger’s first model, at least insofar as its bottom-line analysis concerned the hypothesis of a “shriveling” or “narrowing” of manic temporal experience to “enpresenting.”^23^

By contrast, our data lend support to Minkowski’s first hypothesis. The patterns we identified suggest that there is indeed a distinctive form of temporal experience at work in mania, and that this temporal structure has important consequences – for example, regarding awareness of illness, practical reasoning, and awareness of risk. In order to bring this out more fully, let’s consider the following refinement (and non-metaphorical specification) of Binswanger’s second model. We might call it “the Olga Blum variant,” since it takes its lead from Binswanger’s third deployment of his governing metaphor:

- A core feature of acute mania lies in a *sui generis* form of existential protention of a future with a uniformly positive valence.
- This form of manic protention extends not only to the next few seconds (“the about-to-occur”), but has a global significance, projecting a structure for the self-implicating future as a whole.
- Manic protention is in Binswanger’s distinctive sense “*maßgebende*” [norm-establishing] – that is, it establishes a normative standard that shapes both specific expectations and the interpretation of future experiences.
- Because protention is one of the basic constituents of the lived present, manic protention results in a distinctive form of *Präsenzzeit* [presence-time, lived presence] characteristic of mania.

Reverting to Binswanger’s metaphor, we might say that a *sui generis* form of manic protention is *the dominant thread* in the temporal fabric of manic optimism.

Second-person phenomenology is of necessity indirect and hermeneutic; it is rare to find a definitive proof for a hypothesis. Moreover, there are intrinsic limits in trying to detect a non-presentifying form of futural experience using interview techniques that by their nature invite research participants to engage in explicit, presentifying representations. These limits notwithstanding, we submit that this Olga Blum variant on Binswanger’s second model fits with the data that emerged in our study – and this in two senses. First, the Olga Blum variant fits our data in the modest sense of being consistent with it; as we have seen, Minkowski’s second hypothesis did not fare so well! But more importantly, the Olga Blum variant fits the data in the sense that it helps explain important patterns that emerged in the interviews.

Consider first the patterns regarding participants’ awareness of their own manic condition. As we have seen, participants exhibited detailed awareness of past mania and knowledge of specific warning signs of imminent episodes. Yet they denied both current mania and the likelihood of future episodes. This is an unusual and temporally distinctive form of what in psychiatry is often described as “impaired insight.”^24^ But the Olga Blum variant on Binswanger’s second model makes sense of it. For on that hypothesis, manic experience is dominated by a global protention of an “all-good” future; *future* recurrence of illness resists accommodation, since it conflicts with a norm-establishing (*maßgebende*) protention. What about *present* illness? Recall that on Binswanger’s model, protention plays a decisive role in the *Aufbau* [construction] of the experienced present. A dominant manic protention can thus be expected to yield a form of experienced presence that cannot easily accommodate the thought that something is *wrong*. Since awareness of present illness is *ipso facto* acknowledgement that something is wrong, such awareness stubbornly resists accommodation. Moreover, all participants in our study were keenly aware that *past* episodes of mania had resulted in profoundly negative consequences. An acknowledgement of present illness would bring with it an expectation of future negative consequences. But this conflicts with the dominant protention.

Notice that none of this interferes with the memory of *past* manic episodes, since the core of the hypothesized disturbance concerns a temporally asymmetrical protention. This variant on Binswanger’s hypothesis in this sense fits the data, explaining a striking and otherwise unexplained temporal asymmetry in participants’ awareness-of-illness. It also helps in making sense both of the general descriptions offered by participants of their manic condition (suddenly breaking through to an experience of a “bright” world), and of the distinctive character of their remarks when asked about the future – remarks which were uniformly positive but almost entirely unspecific.

We turn in the final section to consider the explanatory force of this variant on Binswanger’s hypothesis with respect to impairments of inductive reasoning, together with its bearing on Minkowski’s third hypothesis. Before doing so, however, it is worth taking note of the explanatory advantage that our hypothesis enjoys over one natural rival. According to this alternative hypothesis, mania is first and foremost an *affective disorder*, and its core disturbance involves highly elevated affect or mood. On this approach, manic optimism, with its distinctive attitude about the future, is seen as at most a derivative phenomenon. Because elevated mood has a pervasive impact on experience, it is to be expected that a person in a highly elevated mood will experience *everything* (including the future) as good.

In assessing the comparative merits of this alternative, we need first to be clear that we do not deny that elevated affect is an important feature of mania. Both Minkowski and Binswanger advance the hypothesis that temporal disturbance is *a* core feature of mania; we should be open to the possibility that there are other core features. Moreover, we take one of the important insights from the phenomenological traditions (both philosophical and clinical) to be that moods have significant temporal structure. Binswanger’s hypothesis is best understood as a specification of the temporal structure of the affect at work in manic excitement. With these two clarifications in hand, we come to the crucial point vis-à-vis this alternative explanation: the fact is that the participants in our study *did not experience everything as all-good*. They experienced *the future* as all-good, and this in turn had a marked effect on their experience of the lived present. But the *past* which they recollected was *not* all good; on the contrary, it included distinctly negative and painful experiences. A key advantage of Binswanger’s hypothesis is that it explains this asymmetry. Appeal to elevated affect alone (without a supplementary account of its distinctive temporal structure) leaves the asymmetry unexplained.

## 6. Manic temporality and existential logic

We turn finally to consider the bearing of this analysis on impairments of reasoning, and specifically on inductive reasoning, in conditions of manic excitement. There is a classic perspective on inductive reasoning, exemplified by the wartime papers of Rudolph Carnap and Carl Hempel, which treats induction as a strictly formal, wholly syntactic operation.^25^ The merits of that account have been vigorously debated among logicians. But even on this maximally formal approach, issues about time enter the logical picture, albeit only time of a very particular sort and playing a sharply defined role. Carnap’s elusive *c*-function is emblematic:

$$TAPODI={\left[SSTA\right]}_{TA}-{\left[SSTA\right]}_{TEP,}$$

According to this top-level formula, the degree of credence that Jones ought to have for hypothesis *h* is a function of *h* (the hypothesis) and the evidence available to Jones.^26^ But note the occurrence of ‘*t*’ on both sides of the equation. What is at stake is Jones’ credence with respect to *h at time t*; the *c*-function is meant to specify its relationship to the evidence available to Jones *at t*. In other words, the rationality of a particular inductively based belief is always indexed to the evidence available *at the time*. At which time? In effect: at the instant when a bet is placed.

It is crucial to remember, however, that inductive reasoning is not only a matter for formal logic; it is also a phenomenon of lived experience. Considered in this context, induction has a much richer and more complex relation to time. We propose to use the term “existential logic” to refer to the study of reasoning (including inductive reasoning) as a structure of experience. The key point to recognize is that existential logic in this sense is *richly temporal*. As a phenomenon of lived experience, induction is about knitting together the lived past with the lived future. And this holds even if the subject matter of the induction is something long-past. Why? Because a lived induction is always a way of gathering up evidence from past experience and using that to project oneself into the future – whether by making some explicit prediction or as a matter of anticipating what future evidence-gathering will bring. Viewed from this perspective, we should *expect* that serious disturbances to the structure of temporal experience will have consequences for a person’s inductive abilities.

With this context in mind, we return to the data. We have already shown that our variant of Binswanger’s Olga Blum model helps to make sense of an otherwise unexplained pattern of impaired insight manifested by the participants in our study. We are now in a position to see that something similar holds for the pattern of inductive failure. According to our hypothesis, the form of temporal disturbance in mania involves an unspecific but nonetheless dominant protention of global significance and unqualified positive valence. For someone whose temporal experience is characterized by such a disturbance, we should *predict* that lived induction will be impaired in specific ways.

Here it is worth pausing to recall Minkowski’s patient with whom we began. Minkowski’s patient experienced a future which was absolutely dominated by the anticipation of imminent torture and execution. Evidence that conflicted with that dominating experience simply failed to gain traction in inductive proceedings. In mania the disturbance is inverted. On the hypothesis that we have developed here, temporal experience in mania is dominated by a distinctive global protention of unspecified but exclusively good things to come. In the words of one participant:

(14) BP2: Yeah. You feel happy, you feel things are working perfect.

Like all protentions, this distinctive manic protention is determinately indeterminate. In this case, the key moment of determinacy is that everything will be good; everything will work out. Exactly *how* things will work out so well, particularly in light of current behavior that others see as reckless, is left almost entirely undetermined. And as Binswanger proposed in the case of Olga Blum, this *sui generis* form of manic protention “hangs in the air,” untethered by retentions that might temper it.

If indeed temporal experience were distorted in this way, what would be the consequences for “lived induction”? Where inductive evidence points toward a conclusion that some particular matter will *not* be “all-good,” will *not* “work out,” we can expect that evidence to resist inductive incorporation, since it conflicts with the dominant protention. The result would be cognitive dissonance in milder states of mania or, in more severe states, the sense that past experience is irrelevant as a guide to the future. And this is indeed what the data from our study attests. Recall the words of BP9: “This is a new game now.”

What is common between Minkowski’s patient and the participants in our study is, first, a specific disturbance in temporal “unfolding,” and second, an impairment of inductive reasoning. What we can see now is that the former explains the latter. When inductive evidence conflicts with melancholic pessimism, that evidence cannot be effectively taken up. And when inductive evidence conflicts with manic optimism, pointing toward a possible negative outcome, that evidence resists incorporation within a lived induction. One crucial consequence is particularly worth noting: manic temporality threatens a catastrophic loss of vital contact with the reality of a world characterized by risk.

Once again, we can gauge the strength of our proposed analysis by comparing it to a natural rival. We have proposed that a *sui generis* form of protention is a core element of mania, and that this temporal disturbance helps to explain a manifest pattern of failure of inductive reasoning. But might the order of explanation run in the opposite direction? Could we posit a failure of inductive reasoning (or perhaps some more general cognitive failure) as the core feature, and then interpret the disturbance of protention as a consequence?^27^ In considering this alternative analysis, it is important to be clear that the data from our study does not suffice to offer clear evidence about causation. Nonetheless, we submit that the data supports our hypothesis over this natural rival.

The pattern of inductive failure that we identified among participants in the study manifested itself specifically with regard to the likelihood of future manic episodes and negative future outcomes in light of patterns reported from past experience. We encountered no evidence of a general collapse of inductive abilities. The specificity of manifest impairment is left unexplained if we posit either a general cognitive deficit or a global failure of induction as a core feature of mania. Of course one might in principle posit a narrow form of inductive failure as fundamental to mania, but to do so would be problematically ad hoc. A key advantage of our hypothesis is that it *explains* the specificity in the manifest pattern of cognitive deficit by appeal to a specifiable disturbance in a discrete component of temporal experience. For as we have seen, our hypothesis predicts just the sort of inductive impairment that participants manifested: an inability to incorporate inductive evidence that conflicts with the dominant protention.

## 7. Limitations and future research

Our findings are of significance in psychiatry, law, and philosophy. Clinically, our results articulate a core feature of the experience of patients in an acute and understudied phase of illness; they both identify and explain an unusual pattern of temporally asymmetrical impaired insight. Our findings are legally significant in identifying a pattern of impaired reasoning that has direct bearing in applying legal standards of “competence” or “decision-making capacity,” informing strategies for balancing the legal imperative to protect vulnerable persons (on the one hand) and the obligation to support and respect a person’s decisions (on the other). Philosophically, our results suggest a complex interconnection among structures of temporal experience, self-knowledge, and fundamental operations of reason.

Our study exhibits limitations that are common in small-scale, qualitative research. The number of participants made multiple, in-depth interviews and analysis feasible, but precluded statistical analysis. There was no control group, although our analysis was informed by experience using a similar protocol with two populations of persons diagnosed with depression (Owen, Freyenhagen, Hotopf, & Martin, 2015). The research methodology dictated selection of participants characterized by high verbal skills, severe illness, and absence of significant co-morbidities (e.g. addictions, personality disorder); these requirements narrowed the pool of eligibility. And as noted above, reliance on the clinical interview limited our ability to gather evidence as regard the non-presentifying character of manic protention.

The body of research on temporal experience in mania has been growing in recent years, but remains small overall, particularly in comparison to the analogous body of research on depression. Our results contribute to this growing research corpus in at least three ways. First, we have reconstructed significant models of manic temporality from the history of clinical phenomenology. Second, we have demonstrated the relevance for mania of a form of temporal experience (protention) that is distinct both from the experience of passing time and time flow, and from judgements of duration – the phenomena that have been the central focus in recent controlled studies. This in turn suggests (third) that the standardized vocabulary proposed for research in this area (Bschor et al., 2004, p. 223) may need to be revisited.

We conclude by noting two research questions that are raised, but not addressed, by our findings. As noted at the outset, recent controlled studies have demonstrated a difference in the experience of the flow of time between persons with mania and healthy controls: for persons in manic states, time is more often experienced as flowing quickly. Our study provides evidence of a distinctive form of protention at work in manic episodes – an “all-rosy” future is projected. A natural question for further research concerns the possible connections between the two phenomena. We have not addressed this question here, but it is a matter that merits both conceptual and experimental attention. Second, we note that the Olga Blum variant on Binswanger’s second model would predict a further asymmetry in inductive failure during manic episodes. If indeed the distinctive form of protention at work in mania projects an “all-rosy” future, then we should expect inductive failure during manic episodes to manifest itself specifically with respect to future *negative* outcomes. Evidence that inductively warrants a judgement about a future *positive* outcome would not resist incorporation to the same extent. This hypothesis could be tested empirically.^28^

## Notes

1. One tell-tale detail: at one point Minkowski reflects on the patient’s reaction “when I shaved in front of him” (Minkowski, 1923, p. 135).

2. For a reconstruction of this approach to psychopathology, see Berrios (1996).

3. On the use of the form-content distinction in the history of clinical phenomenology, see Stanghellini and Aragona (2016).

4. For Minkowski’s somewhat uneasy appropriation of the term, see Minkowski (1933, pp. 230–231).

5. According to Herbert Spiegelberg, Minkowski’s knowledge of Husserl was limited to the *Logical Investigations* and the 1929 *Paris Lectures*, at least part of which he seems to have attended in person (Spiegelberg, 1972, p. 238). Minkowski seems never to have engaged seriously with Heidegger’s writings.

6. There is, admittedly, an element of anachronism in this way of putting it, since Minkowski published his three hypotheses about mania only *after* Binswanger’s first model was developed. However, there is also an element of truth in the anachronism, as even Binswanger’s earlier model is punctuated at key points with references to Minkowski’s work on temporality and mood disorder. Binswanger almost certainly had knowledge of Minkowski’s “suggestions” about mania prior to their publication in *Lived Time*.

7. Translations from this text are our own. The material originally appeared in Vols. 27–30 of the *Schweizer Archive für Neurologie und Psychiatrie.*

8. For a discussion of this Heideggerian principle, see Smith (1981, pp. 211–235).

9. Heidegger (1927, H346–H349). The German term is Neugier – literally: greed or lust for the new.

10. Macquarrie and Robinson translate *Aufenthaltlosigkeit* with the compound phrase: “never-dwelling-anywhere.” Stambaugh translates it as “the inability to stay at all.” For present purposes, we shall translate the term as “restlessness.”

11. For Heidegger’s description of the curious person as “*überall und nirgends,”* see Heidegger (1927, H347).

12. The culminating sentence of Binswanger’s temporal analysis carries a footnote (Binswanger, 1933, p. 162n) that refers back to a number of sources, the first of which is Minkowski’s 1923 study of his severely depressed patient.

13. Binswanger is here adapting Heidegger (1927, H347). The term *Augenblick* is a term-of-art in *Being and Time*; Macquarrie and Robinson translate it rather freely as “moment-of-vision.”

14. A full discussion of these Heideggerian doctrines falls beyond the scope of the present article. For one relevant piece of textual evidence, see Heidegger (1927, H359–H360), where Heidegger claims that “circumspective enpresenting [*Gegenwärtigung*] … *always belongs to a full ecstatical unity of temporality*” and goes on to describe the present as “rooted in the future and in having-been.” (translation and emphasis altered). At H326, he describes the future as “releasing from itself the present.” These claims require greater scrutiny than is possible here, but on one reading they would seem to preclude the possibility of temporal experience that is “shriveled to mere enpresenting.”

15. See Husserl (1950, vol. X, p. 21). For Stern’s use of “*Präsenzzeit*,” see Stern (1897). On the controversies surrounding the sources of Husserl’s notion of *Präsenzzeit*, and on the history of its (mis)translation, see Gallagher (1998, chap. 3).

16. For the mistranslation, see Husserl (1964), p. 41. The duration of the specious present has been the subject of considerable debate, but it is generally agreed that the relevant units are seconds or milliseconds (Pockett, 2003).

17. The extension of the Husserlian concepts of protention and retention to longer timescales has recently been discussed in Kupke (2009, p. 40).

18. See Binswanger (1960); translations from this text are our own. See also Binswanger (1964).

19. The term *Aufbau* literally means “build-up”; more colloquially it translates “construction” or “structure.”

20. ‘*Maßgebend-*’ is another technical term that resists straightforward translation. The standard dictionary definition is “definitive” or “authoritative”; etymologically the sense is something like: “giving the measure,” or “establishing the standard.” The corresponding noun, ‘*Maßgabe*’ means “stipulation”; ‘*Maßstab*’ is the ordinary term for a yardstick or ruler. So perhaps the best translation of ‘*maß-gebenende*’ into contemporary philosophical English would be “*normative*.” Something is *maßgebende* in Binswanger’s sense insofar as it establishes a standard by which other experience is assessed, shaping both specific expectations and the interpretation of other experiences. We leave the term untranslated, but gloss it as “norm-establishing.”

21. Interview questions included: “In terms of your spirits and mood right now, how would you describe it?,” “If you have a sense of how things are now and your future, how does the future feel?.”

22. One might worry here that the interviewer explicitly asked about the participants’ future and past and, therefore, may have acted in a suggestive way. By way of clarification, we would emphasize first that the questions posed by the interviewer were designed to be open rather than leading. That they were indeed open questions is confirmed by the fact that use of a similar interview schedule elicited quite different replies about the future in a related study focusing on persons diagnosed with severe depression (Owen et al., 2015). Finally, we note that BP9’s remark about plans to renew a television license (see extract 5, above) was made independently of any interview question about the future, being offered spontaneously at the end of a nearly 300-word response to a question about the differences between the participant’s experiences inside and outside of hospital. In the course of this wide-ranging response, BP9 discussed past indebtedness, subsequent struggles to get support to cope with credit card bills, current solvency and employment, as well as the plan to pay for the television license. These responses simply cannot be reconciled with the hypothesis of “living only in the now” – whatever the prompt that elicited them. (We thank an anonymous referee for raising this possibility, and giving us the opportunity to add this clarification.)

23. The mismatch between our data and Minkowski’s second hypothesis is so striking that it calls out for an error theory of some sort – an explanation of how Minkowski went so far wrong on this point. We do not offer such an explanation here, but it is certainly worth reconsidering in this context Minkowski’s uneasy reliance on Mignard’s notion of “subduction,” which projects an understanding of mental illness as some kind of reduced form or “lower level” of experience. The power of such metaphors can be considerable in shaping hypothesis-generation in psychiatry and phenomenology alike.

24. For analysis of the clinical notion of insight, see Amador and David (2004).

25. See Hempel (1942) and Carnap (1945). For a useful synopsis, see Jeffrey (1973).

26. We adapt this version of the formula from Jeffrey (1973, pp. 299–300).

27. We are grateful to an anonymous referee for raising this possibility.

28. Support for the research presented here was provided by a grant from the Wellcome Trust (grant number 097683/Z/11/Z). The authors thank the participants in the study for making this research possible. Earlier versions of the article were presented at the American Society for Existential Phenomenology, The Essex Phenomenology *Werkstatt*, and the Kings College London Philosophy and Medicine Colloquium. For helpful comments on earlier drafts, the authors would like to thank these audiences, and two anonymous referees for this journal.

## References

[CIT0001] Amador X., & David A. (Eds.). (2004). Insight and psychosis: Awareness of illness in schizophrenia and related disorders, 2nd ed. Oxford: Oxford University Press.

[CIT0002] Berrios G. (1996). *The history of mental symptoms: Descriptive psychopathology since the 19th century*. Cambridge: Cambridge University Press.

[CIT0003] Binswanger L. (1933). *Über Ideenflucht [On the flight of ideas]*. Zurich: Fuessli.

[CIT0004] Binswanger L. (1946). Über die daseinsanalytische Forschungsrichtung in der Psychiatrie [The existential-analytic research program in psychiatry]. *Schweizer Archive Für Neurologie Und Psychiatrie*, 57, 209–225. 20276957

[CIT0005] Binswanger L. (1960). *Melancholie und Manie: Phänomenologische Studien [Melancholy and mania: Phenomenological studies]*. Pfullingen: Neske.

[CIT0006] Binswanger L. (1964). On the manic mode of being-in-the-world Straus E. (Ed.), *Phenomenology pure and applied*. Pittsburgh: Duquesne University Press 127–141. Reprinted in M. Broome, R. Harland, G. Owen, & A. Stringaris (Eds.), *The Maudsley reader in phenomenological psychiatry* (pp. 197–203). Cambridge: Cambridge University Press, 2012.

[CIT0007] Blattner W. (1999). *Heidegger’s temporal idealism*. Cambridge: Cambridge University Press.

[CIT0008] Bschor T., Ising M., Bauer M., Lewitzka U., Skerstupeit M., Müller-Oerlinghausen B., & Baethge C. (2004). Time experience and time judgement in major depression, mania and healthy subjects: A controlled study of 93 subjects. *Acta Psychiatrica Scandinavica*, 109, 223–229. 10.1046/j.0001-690x.2003.00244.x 14984395

[CIT0009] Carnap R. (1945). The logic of induction. *Philosophy of Science*, 12(2), 72–97.

[CIT0010] Charmaz K. (2006). *Constructing grounded theory: A practical guide through qualitative analysis*. London: Sage Publications.

[CIT0011] Dennett D. (1992). *Consciousness explained*. Boston: Little, Brown and Co.

[CIT0012] Gallagher S. (1998). *The inordinance of time*. Evanston, IL: Northwestern University Press.

[CIT0013] Glaser B., & Strauss A. (2017). *Discovery of grounded theory: Strategies for qualitative research*. New York: Routledge.

[CIT0014] Guerlac S. (2006). *Thinking in time: An introduction to Henri Bergson*. Ithaca: Cornell University Press.

[CIT0015] Heidegger M. (1927). *Sein und Zeit [Being and time]*. Tübingen: Niemeyer Except where indicated, we follow the Macquarrie and Robinson translation (*Being and time*, New York: Harper and Row, 1962); page references are given to the usual H pagination of the first edition.

[CIT0016] Hempel C. (1942). A purely syntactical definition of confirmation. *The Journal of Symbolic Logic*, 8(4), 122–143.

[CIT0017] Husserl E. (1950). *Husserliana*. The Hague: Martinus Nijhoff.

[CIT0018] Husserl E. (1964). *The phenomenology of internal time-consciousness. (J. Churchill)*. Bloomington, Indiana: Indiana University Press.

[CIT0019] Jeffrey R. (1973). Carnap’s inductive logic. *Synthese*, 25(3/4), 299–306.

[CIT0020] Kortooms T. (2002). *Phenomenology of time: Edmund Husserl’s analysis of time-consciousness*. Dordrecht: Kluwer.

[CIT0021] Kupke C. (2009). *Der Begriff Zeit in der Psychopathologie [The concept of time in psychopathology]*. Berlin: Parodos Verlag.

[CIT0022] Mahlberg R., Kienast T., Bschor T., & Adli M. (2008). Evaluation of time memory in acutely depressed patients, manic patients, and healthy controls using a time reproduction task. *European Psychiatry*, 23, 430–433. 18515048 10.1016/j.eurpsy.2007.07.001

[CIT0023] Mezey A., & Knight E. (1965). Time sense in hypomanic illness. *Archives of General Psychiatry*, 12, 184–186. 14237628 10.1001/archpsyc.1965.01720320072008

[CIT0024] Mignard M. (1924). La subduction mentale morbide et les théories psychophysiologiques [Morbid mental subduction and psycho-physiological theories]. *L’Année Psychologique*, 25, 85–105.

[CIT0025] Miller I. (1984). *Husserl, perception and temporal awareness*. Cambridge, MA: MIT Press.

[CIT0026] Minkowski E. (1923). Étude psychologique et analyse phénoménologique d’un cas de mélancolie schizophrénique, [Psychological study and phenomenological analysis of a case of schizophrenic melancholia]. *Journal De Psychologie Normale Et Pathologique*, 20, 543–558. Citations refer to the pagination of the translation by Bliss, B: “Findings in a Case of Schizophrenic Depression,” in May, R., Angel, E. & Ellenberger, H. (Eds.). *Existence: A new dimension in psychiatry and psychology*. New York: Simon and Schuster, 1958, 127–138.

[CIT0027] Minkowski E. (1933). *Le temps vécu: Etudes phénoménologiques et psychopathologique*. Paris: Delachaux Collection de l’Evolution Psychiatrique Citations refer to the pagination of the translation by Metzel, N.: *Lived time: phenomenological and psychopathological studies*. Evanston, Illinois: Northwestern University Press 1970.

[CIT0028] Owen G., Freyenhagen F., Hotopf M., & Martin W. (2015). Temporal inabilities and decision-making capacity in depression. *Phenomenology and the Cognitive Sciences*, 14(1), 163–182.

[CIT0029] Owen G., Freyenhagen F., & Martin W. (2018). Assessing decision-making capacity after brain injury: A phenomenological approach. *Philosophy, Psychiatry and Psychology*, 25(1), 1–19.

[CIT0030] Owen G., Freyenhagen F., Martin W., & David A. (2017). Clinical assessment of decision-making capacity in acquired brain injury with personality change. *Neuropsychological Rehabilitation*, 27(1), 133–148. 26088818 10.1080/09602011.2015.1053948 PMC5080972

[CIT0031] Pockett S. (2003). How long is ‘now’? Phenomenology and the specious present. *Phenomenology and the Cognitive Sciences*, 2, 55–68.

[CIT0032] Smith Q. (1981). Heidegger’s theory of moods. *The Modern Schoolman*, 58(4), 211–235.

[CIT0033] Spiegelberg H. (1972). *Phenomenology in psychology and psychiatry*. Evanston, IL: Northwestern University Press.

[CIT0034] Stanghellini G., & Aragona M. (2016). Phenomenological psychopathology: Toward a person-centered hermeneutic approach in the clinical encounter In Stanghellini G. & Aragona M. (Eds.), *An experiential approach to psychopathology* (pp. 1–43). Berlin: Springer.

[CIT0035] Stern W. (1897). Psychische Präsenzzeit [Psychic presence-time]. *Zeitschrift Für Psychologie Und Physiologie Der Sinnesorgane*, 13, 325–349.

[CIT0036] Tysk L. (1984). Time perception and affective disorders. *Perceptual and Motor Skills*, 58, 455–464. 6739243 10.2466/pms.1984.58.2.455

[CIT0037] Zahavi D. (2003). *Husserl’s phenomenology*. Stanford: Stanford University Press.

